# Integrated vector management: The Zambian experience

**DOI:** 10.1186/1475-2875-7-164

**Published:** 2008-08-27

**Authors:** Emmanuel Chanda, Fred Masaninga, Michael Coleman, Chadwick Sikaala, Cecilia Katebe, Michael MacDonald, Kumar S Baboo, John Govere, Lucien Manga

**Affiliations:** 1 National Malaria Control Programme, Ministry of Health, Zambia; 2 World Health Organization, WHO Country office, Zambia; 3 Medical Research Council, Durban, South Africa; 4 United States Agency for International Development, Washington, DC, USA; 5 University of Zambia, School of Medicine, Zambia; 6 World Health Organization, WHO ICST, Zimbabwe; 7 World Health Organization, WHO AFRO, Congo; 8 Liverpool School of Tropical Medicine, Pembroke Place, Liverpool, L3 5QA, UK

## Abstract

**Background:**

The Zambian Malaria Control Programme with the Roll Back Malaria (RBM) partners have developed the current National Malaria Strategic Plan (NMSP 2006–2011) which focuses on prevention based on the Integrated Vector Management (IVM) strategy. The introduction and implementation of an IVM strategy was planned in accordance with the World Health Organization (WHO) steps towards IVM implementation namely Introduction Phase, Consolidation Phase and Expansion Phase.

**Achievements:**

IVM has created commitment for Legal and Regulatory policy review, monitoring, Research and a strong stewardship by the chemical suppliers. It has also leveraged additional resources, improved inter-sectoral collaboration, capacity building and enhanced community participation which facilitated a steady scaling up in coverage and utilisation of key preventive interventions. Thus, markedly reducing malaria incidence and case fatalities in the country.

**Conclusion:**

Zambia has successfully introduced, consolidated and expanded IVM activities. Resulting in increased coverage and utilization of interventions and markedly reducing malaria-related morbidity and mortality while ensuring a better protection of the environment.

## Background

In 1992, the Global Strategy for Malaria Control was adopted in Amsterdam as a response to the increasing global malaria burden, which is now responsible for about 515 million cases and one million deaths annually [1-3]. The strategy was founded on four technical elements, which included early diagnosis and prompt treatment of malaria, planning and implementation of selective and sustainable preventive measures including vector control, early detection, containment or prevention of epidemics, and, strengthening of local capacities in basic and applied research. Implementation of this global strategy for malaria control began in Africa in 1993 with the development of national action plans as recommended by a WHO Study Group [4,5].

However, in most parts of Africa, the initial national action plans derived from the strategy had inadequately developed vector control components. As a result, progress in the implementation of vector control activities remained very limited [6]. Some of the factors for the limited vector control implementation included health sector reforms and decentralization, insufficient guidance on vector control implementation, low priority to vector control with insufficient resource allocation, dismantled infrastructure, lack of technical competencies exacerbated by the high vector control staff attrition, and, limited cost-effective technical options. In addition, there were growing concerns over the use of indoor residual spraying with DDT for malaria vector control [7].

The Roll Back Malaria (RBM) initiative that was launched in 1998 was built on the technical elements of the global strategy for malaria control [8]. RBM implementation did not effectively address all the above challenges to vector control, but focused on the promotion of insecticide-treated nets (ITNs) as the main, and in most cases, only, preventive measure [9]. Meanwhile, Ministries of Health continued to face health systems challenges that seriously constrained the implementation of effective malaria vector control.

A new approach was needed to revive vector control. Such an approach was to resolve not only technical issues pertaining to vector control implementation, but also, to take into consideration health systems constraints as well as environmental concerns related to the judicious use of insecticides. Since 2001 the World Health Organization has been promoting Integrated Vector Management (IVM) as the new strategic approach to vector control [10,11]. IVM is defined as the targeted use of different vector control methods alone or in combination to prevent or reduce human-vector contact cost-effectively, while addressing sustainability issues.

IVM should be environmentally sound, inter-sectoral, selective, targeted, cost-effective and sustainable. Two points need to be emphasized, first; IVM is based on the premise that effective control is not the sole preserve of the health sector but requires the collaboration of various public and private agencies and the participation of the communities. Secondly, IVM emphasizes capacity building at the district and municipal level to plan, implement, monitor and evaluate these vector control operations.

A number of countries, including Zambia, have already adopted this approach. Over the past two decades, malaria incidence has risen from 121.5 cases per thousand in 1976 to about 394 cases per thousand in 2001 [12]. In 2002, recognizing the magnitude of this malaria burden, the Government of Zambia adopted a new National Malaria Treatment and Control Policy, with IVM adopted as a key strategic approach to vector control. Implementation of the National Malaria Policy commenced in 2003.

This paper reports on the IVM processes, achievements and the status of key elements of IVM over the past five years.

### The IVM strategy development and implementation

In 2003, the NMCP was refocused to implement a combination of interventions [13] and re-launched IRS in five districts with existent ITNs programmes. Furthermore, in April 2004 Zambia submitted the Round Four Global Funds to fight Aids, Tuberculosis and Malaria (GFATM) proposal in order to scale up effective strategies to reduce malaria-related morbidity and mortality in line with national and global goals [14].

IVM was among the key areas that constituted the malaria component of the GFATM proposal. The objectives and key service areas pertinent to IVM were as follows: (1) reduce malaria transmission in rural and urban districts through; (2) scale up coverage for IRS; (3) expand environmental management and larviciding; (4) increase access and availability of ITNs among pregnant women and children under five years of age; (5) strengthen coordination and partnership development for malaria prevention and control; and (6) improve capacity for monitoring and evaluation [13,14].

The IVM National Strategy was introduced and implemented in accordance with the WHO steps, namely: Introduction Phase; Consolidation Phase and Expansion Phase [15,16] (Table 1). Five districts were selected for the re-introduced IRS programme and 10 RBM sentinel districts for ITNs. The selection criterion of the districts was based on the following factors: (1) Functionality of district health structures; (2) Ongoing vector-borne disease control activities; and (3) Potential for inter-sectoral collaboration with partners and local NGOs.

**Table 1:** Integrated Vector Management (IVM) implementation phases

**Phase**	**Activities**	**Purpose**	**Time**
**Introduction**	Designation of national focal point	To spearhead IVM	October 2004
	Training of national focal point	To develop the National Action Plan	November 2004
	National steering committee constituted	To review and adopt WHO guidelines for VCNA	February 2005
	Vector Control Needs Assessment	To determine gaps in policies, legislation and capacity	May 2005
	National Stakeholders Consensus Workshop	To adopt VCNA report and endorse IVM as national strategy	June 2005
	Draft country specific IVM guidelines produced	To adapt strategies to local conditions	2005
	Capacity building workshops on IVM conducted	To train provincial and district personnel	2005
	National Entomology laboratory upgraded	To enhance evidence-based decision-making	2005
	Five district selected to conduct epidemiological assessments and develop district action plan	To operationalize IVM (IRS, ITNs, Larviciding and E. management).	2005
**Consolidation**	Quarterly national IVM committee meetings conducted	To ascertain functionality of IVM structures	Every quarter
	Yearly review of the national action plan	To incorporate and address policy and regulatory issues	Yearly
	Conduct trainers of trainers workshops	To strengthen capacity for vector control	Yearly
**Expansion**	IVM training workshop conducted	To scale up interventions to 19 districts	November 2006

The selected districts conducted geographical reconnaissance, epidemiological, entomological, and ecological, feasibility assessments and elaborated district specific IVM operational plans as part of the district plan [16-20].

### Status of the five key elements of IVM

#### Advocacy, social mobilization and legislation

The Ministry of Health with the national Roll Back Malaria (RBM) partnership has developed a new National Malaria Strategic Plan (NMSP 2006–2011) whose vision is *"A malaria free Zambia*". The key components of NMSP are: (1) Policy; (2) Partnership coordination: (3) Equity and increased access to malaria control interventions; (4) Strengthening of the health systems for scale-up of key preventive interventions based on the IVM strategy with monitoring; and (5) Evaluation of evidence-based and cost-effective package of interventions with strong collaboration of various public and private agencies that impinge on vector breeding, such as agriculture and urban development including local authorities and engagement of communities with strengthened education, information and communication system [20,21].

Suitable legal and regulatory policy framework exists in country that provide a basis for delivery of IVM in Zambia including, Public Health Act Chapter 295 and Mosquito Extermination Acts (CAP 312) that have been systematically reviewed [22].

The prioritization of malaria within the Basic Health Care Package and its declaration as a public health problem within the National Health Strategic Plan led to increased government and partner commitment to malaria control and enhanced mobilization of resources. In 2003, the Zambian government removed all forms of taxes and tariffs on ITNs and insecticides specific to malaria control demonstrating its commitment to malaria prevention and control [23-25].

Enhanced community participation is an integral component of IVM in Zambia. Spray operators for IRS and applicators for larviciding as well as health workers supporting ITN delivery are drawn from the local communities. To ensure quality, Spray operators are selected based on eligibility criteria that include minimum academic qualification. They undergo an intensive 21 days training on insecticide application and environmental safeguards. Each spraying gang has a team leader and every four spraying teams are supervised by a trained Environmental Health Officer.

#### Collaboration within the health sector and partners

All possible options of intra- and inter-sectoral collaboration have been considered in order to establish a fruitful public and private mix in the delivery of IVM. The Ministry of Health has taken a leading coordination role coordinating communication between the public, private and civil sectors. To this effect, the NMCP and RBM partnership have developed a malaria communication strategy to facilitate the dissemination of key information on all aspects of malaria including vector control [26].

The IVM working group has broadened membership over the past five years to include representation from public sector, research institutions, higher learning institutions, local authorities, private sector and multi-/bilateral development partners. A working group on DDT under the National Implementation Plans (NIPs) has also been established to ensure adherence to the Stockholm convention on DDT usage. Each partner has a role to play in IVM (Table 2).

**Table 2:** Major stakeholders, partners and respective responsibilities

**Major Stakeholders**	**Name of Organization**	**Responsibilities**
**Government, Bilateral and Multilateral Organizations**		
Ministry of Health	National Malaria Control Centre (NMCC)	Plans, coordinates, monitors and evaluates activities
	Provincial Health Office	Plans activities and monitors implementation
	District Health Office	Provides staff, transport, mobilizes partners and conducts implementation
	Medical Stores Limited	Distributes commodities
Bilateral/Multilateral Organizations	USAID, WHO, HSSP, JICA, MACEPA, IVCC, MTC	Provides logistical and technical support, conducts monitoring and evaluation
Academic/Scientific institutions	TDRC, UNZA	Provides technical support and monitoring
Ministry of Defence	Zambia Army	Provides transport and staff
Ministry of Environment	Environmental Council of Zambia (ECZ)	Regulates, insecticide storage and judicious use
Ministry of Housing and local government	Local authorities (Municipal councils)	Provides staff, transport and implements interventions
**Private Sector**				
Mining and Agricultural Companies	Konkola Copper Mines, Mopani Copper Mines, Kansenshi Copper Mines, Bwana Mkubwa Mine and Chambishi Mines, Nakambala Sugar Plc	Technical assistance on capacity building, provides additional workforce, supplements governments efforts by increasing coverage of interventions
Chemical Companies	AVIMA, BAYER, ECOMED, SYNGENTA, CHEMTALK, CROPPACK, Hudson Manufacturing Company	Provides stewardship, transportation, disposal of empty sachets, Technical assistance on capacity building
Collaborative public-private sector partnerships	Valent Biosciences Corporation	Supports operational research on larval source management
**Non Governmental Organization (NGOs)**		
Social Marketing Organization	Society for Family Health	Spearheads social marketing of ITNs
Non Profit making Organization	Zambia Malaria Foundation	Coordinates ITNs distribution by NGOs
**Community**		
Community members	Community Health Workers	Implementation of activities

There is also strong adherence to principles of subsidiarity in planning and decision-making. For example, the development and implementation of national plans is based on a consultative process to assure ownership and participatory autonomy through interaction of community and district level structures.

#### Integrated approach

The main interventions under the IVM strategy are Indoor Residual Spraying (IRS) and Insecticide Treated Nets (ITNs) implemented in eligible urban and rural areas respectively. Larviciding and environmental management (canalization, draining and land filling) are implemented in collaboration with the local authorities and communities as supplementary interventions [12] (Table 3).

**Table 3:** Total coverage of IVM interventions implemented from 2003 to 2007

**Intervention**	**Name of Commodities/** **approach**	**Total ** **Commodities**	**Total Cost** ** US$**	**Targeted** ** Districts**
Indoor Residual House Spraying	Insecticides; DDT and Pyrethroids (Kgs)	270,392	12,811,127	15
Insecticide-Treated Nets (ITNs)	Insecticide Treated Nets (ITNs)	5,307,142	18,467,000	57
Larviciding	Larvicides (Abate) used (ltrs)	13,802	565,271	5
Environmental management	Dambo drained/canalised(Km^2^)	456	246,760	5

The main objectives of the ITN and IRS programmes are, to ensure that at least 80% of people sleep under an ITN in every district of the country and to have at least 85% of people sleep in sprayed structures in eligible areas of selected districts by 2008 and maintained to 2011 respectively.

In order to explore synergies and to ensure integration of vector control activities an IVM committee was established in the MOH that addresses priority issues on implementation, capacity building, financing and monitoring and evaluation. The integration with other vector-borne disease control, schistosomiasis and trypanosomiasis, in Zambia will be encouraged through the IVM committee.

#### Evidence-based decision-making

Prior to the implementation of interventions, districts conducted geographical, epidemiological, entomological and ecological, feasibility assessments to ensure that methods are based on knowledge of factors influencing local vector biology, disease transmission and morbidity [17-19,27]. In order to make evidence based decisions on vector control systematic studies on vector bionomics and monitoring resistance to insecticides currently in use for public health [27-29] will become part of the IVM. Including contact bioassays to determine both the residual efficacy of insecticides and the quality of spraying.

Research towards the adoption of viable alternatives to chemical insecticide-based vector control methods is being investigated [18,19]. As part of the IVM, alternatives such as the bio-larvicide *Bacillus thuringensis israelensis *(Bti) for larviciding are being investigated [30]. For effective monitoring and evaluation of IVM activities, indicators and methodologies have been defined using WHO-AFRO generic guides, which have been included in the national action plan [16]. Guidelines and checklists for various interventions such as ITNs, IRS etc have been developed to facilitate the routine monitoring and evaluation of the programmes.

#### Capacity-building

Successful IVM is evidenced based maximizing the utilization of available infrastructure and resources, and integrates all available and effective interventions. A central requirement to this is a team of people that have the required skills to develop and manage the IVM [31,32]. Building this capacity in the vector control services is an ongoing process and is occurring at all levels from national down to the community. Currently nine provincial and 44 district environmental officers have been trained on IVM [33] with further expansion of this core group of people planned.

A vector control unit was established at the NMCP with postgraduate level staff. The Unit organized capacity building workshops (training of trainers) for provincial and district level personnel (Environmental Health Technicians, Programme Managers, Coordinators and Supervisors). These were followed by annual district training for DHMTs, local authorities and communities, with the technical support drawn from public sector and private sector partners.

## Results

Feasibility assessments in IVM pioneering districts confirmed the occurrence of three major afro-tropical vectors of malaria; *Anopheles gambiae s.s, Anopheles arabiensis *and *Anopheles funestus *in varying proportions [34,35]. Baseline insecticide susceptibility tests conducted on the three species with DDT (4%), Deltamethrin (0.05%), Alpha-cyhalothrin (0.05%), Malathion (5%) and Propoxur (0.1%), according to WHO standard protocol [36], showed 100% susceptibility.

**Figure 1:**
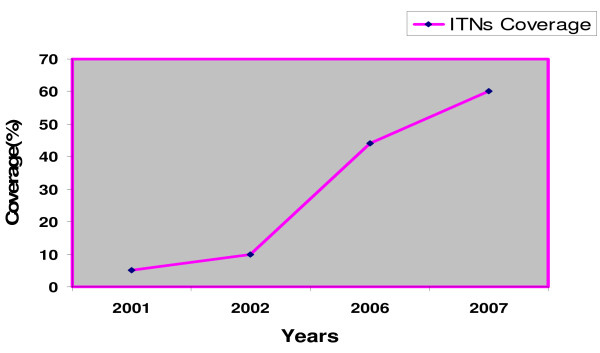
Coverage of insecticide-treated nets from 2001 – 2007.

**Figure 2:**
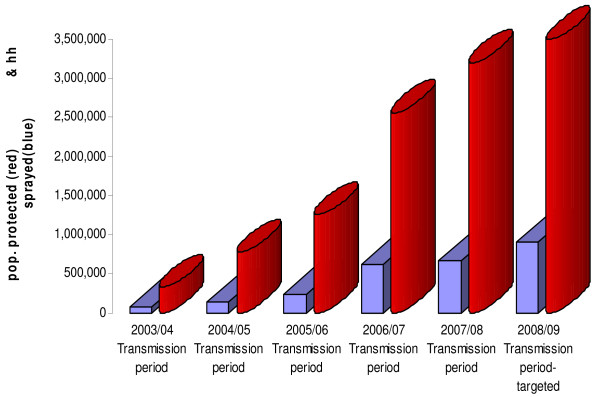
**IRS coverage and population protected from 2003 to 2007 with projections for 2008**.

From 2003 to 2007 about 5,307,142 nets have been distributed (Figure 1), using the Ant-natal, Mass (free of charge) and Commercial distribution mechanisms [12]. In addition, over one million retreatment kits have been distributed during annual net retreatment campaigns during the Child Health Weeks and malaria commemorative days. The 2001 and 2007 national Demographic Health Surveys and the 2004 Roll Back Malaria Surveys show that the number of households with at least one ITN has increased from 27% in 2001 to 40.1% in 2004 and 53% in 2007. In the same years, net utilization has improved markedly from 17.0% to 24.2% and 28.6% in children under the age of five and from 17.7% to 25.1% and 32.7% in pregnant women respectively [37-39]. Equally, the number of structures sprayed has steadily increased from 69,774 in 2003, 178,554 in 2004, 424,000 in 2005, and 537,877 in 2006 to 657,695 in 2007. The average operational coverage in the respective years was 91.4%, 88.2%, 83.5%, 86.7% and 93.5% thus protecting 342,137, 771,644, 1,163,802, 2,160,655 and 3,286,514 people accordingly (Figure 2).

**Figure 3:**
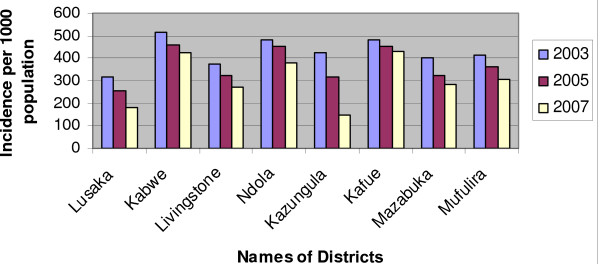
Malaria incidences in IRS districts.

**Figure 4:**
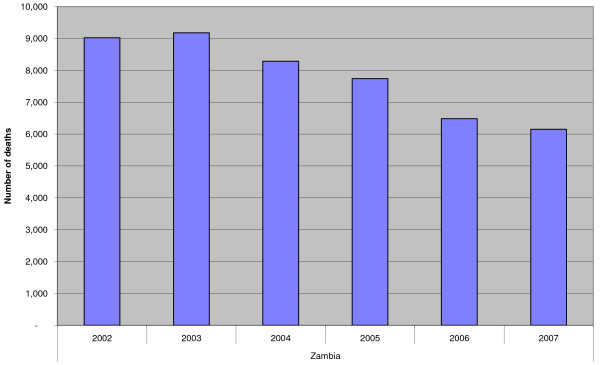
**Trends of malaria deaths from 2002 – 2007**.

The Health Information Management System (HMIS) data [40] indicate that the incidence of malaria in IRS implementing districts has reduced markedly in the last five years as shown in Figure 3. The national incidence of malaria per thousand population has declined from 424.0 in 2003 and 374.3 in 2005 to 358.0 in 2007. Correspondingly, the trends of malaria deaths exhibit substantial reduction as depicted in Figure 4. The RBM and NMCP survey reports have as well demonstrated that parasite rates declined in children between two and nine (2–9) years from 20.1% to 7.4% in 2004 and 2006 respectively [38,41].

## Discussion

Zambia has successfully introduced, consolidated and expanded IVM activities in a step-wise manner according to WHO guidelines [16]. The introductory phase of IVM, marked significant milestones which included capacity building, recruitment of IVM focal point, training in IVM skills development of the national IVM framework with technical support from WHO. Other milestones include: (1) National Steering Committee, Vector Control Needs Assessment; (2) National Strategic Framework and IVM Technical Guidelines; (3) Availability of a core of human and technical capacity for IVM implementation; (4) Selection of districts with high malaria endemicity for IVM activities based on geographical, epidemiological, entomological and ecological information; and (5) Feasibility assessments specific districts for IVM operational plans as part of the overall strategic plan [23,31].

During the consolidation phase, functional IVM structures including the IVM Coordination Committee and a National Steering committee that included major stakeholders were established. These committees address broad policy and regulatory issues including; the mosquito extermination act, Public Health Act, pesticides, role of IVM in the implementation and the Stockholm convention on Persistent Organic Pollutants (POPs). The committees also adopted a joint programme for planning and annual reporting mechanisms for funding.

Through the adoption of this programme an environment for implementing and scaling up IVM exists within the NMCP/MoH. This includes a six-year National Strategic Plan with coordination mechanisms within and outside the MoH to build on the current [15] districts that have on-going IVM.

The National IVM Coordinating Committee has supported a review of relevant statutory instruments and ensuring their enforcement. Though the entomology laboratory has been strengthened, there is still need to strengthen evidence-based implementation of IVM activities through enhancement of systematic studies on vector bionomics and their susceptibility to insecticides at district level. Human resource capacity strengthening will continue to be a priority during the IVM Expansion Phase and vector-borne disease still remains a major challenge.

In the past five years, deployment of a combination of transmission-reducing interventions, in addition to an effective case management, has recorded enormous achievements in Zambia. The introduction of the IVM strategy has not only progressively improved the coverage and utilization of IRS and ITNs, but culminated in an appreciably marked reduction of malaria-related morbidity and mortality. Thus, clearly demonstrating that implementation of vector control interventions in the context of the IVM strategy with adherence to all the five key elements of the approach, as recommended by World Health Organization [42], is very effective for malaria control.

## Conclusion and way forward

Zambia has successfully implemented IVM activities in accordance with the WHO recommended steps towards implementation. Enhanced advocacy, social mobilization and the availability of legislation has greatly stimulated community awareness, culminating into community participation in the delivery of key preventive interventions.

A needs assessment has facilitated the development of an evidenced-based IVM strategy, which in turn has facilitated the strengthening of effective vector control in the country.

Availability of IVM structures at district level has enabled the districts to plan, budget and produce annual reports on IVM activities. Additionally, the establishment of National IVM Working Group facilitated the development of country specific IVM guidelines, information, education and communication materials and, inter-sectoral collaboration with several partners (e.g., private sector, NGOs, local authorities and line ministries). The strategy has also created a platform to address broad policy and regulatory issues and leveraged additional resources. The resources have been used in priority activities such as capacity building of district-provincial personnel in IVM techniques. Thus, there is a steady scaling up of preventive interventions and an increased monitoring and evaluation.

Though IVM has ensured rational utilization of available resources through integration of chemical and non-chemical approaches and, research towards viable alternative interventions. There is now a need for integration with other disease control programmes.

Despite increased progress in implementing IVM in Zambia, there is need to develop methodologies that demonstrate the impact of IVM on the spatial and temporal bionomics of the vectors and their resistance status and further reduction of malaria in Zambia which remains a major challenge.

The implementation of the IVM strategy in Zambia has contributed to the strengthening of malaria vector control, through improved efficiency and capacity development. Thus, markedly reducing malaria-related morbidity and mortality, in addition to a better outcome for the protection of the environment

## Competing interests

The authors declare that they have no competing interests.

## Authors' contributions

EC participated in the IVM strategy development, drafted the manuscript and finalized the analysis, FM contributed to the draft manuscript, MC corrected the manuscript and guided analysis, CS and CK implemented interventions and provided statistics, MMcD, KSB and JG contributed to the drafting and revising of the manuscript and LM conceived the initial ideas and contributed to the drafting of the paper. All authors read and approved the final manuscript.
